# Estimation of stress distribution in ferromagnetic tensile specimens using low cost eddy current stress measurement system and BP neural network

**DOI:** 10.1371/journal.pone.0188197

**Published:** 2017-11-16

**Authors:** Jianwei Li, Weimin Zhang, Weiqin Zeng, Guolong Chen, Zhongchao Qiu, Xinyuan Cao, Xuanyi Gao

**Affiliations:** 1 School of Mechanical Engineering, Beijing Institute of Technology, Beijing, China; 2 College of Mechanical and Electrical Engineering, Henan Agricultural University, Zhengzhou, China; 3 Beijing Carduo Information Technology Company Limited, Beijing, China; 4 School of Mechanical and Electrical Engineering, Shandong Management University, Jinan, China; 5 School of Information and Electronics, Beijing Institute of Technology, Beijing, China; Chongqing University, CHINA

## Abstract

Estimation of the stress distribution in ferromagnetic components is very important for evaluating the working status of mechanical equipment and implementing preventive maintenance. Eddy current testing technology is a promising method in this field because of its advantages of safety, no need of coupling agent, etc. In order to reduce the cost of eddy current stress measurement system, and obtain the stress distribution in ferromagnetic materials without scanning, a low cost eddy current stress measurement system based on Archimedes spiral planar coil was established, and a method based on BP neural network to obtain the stress distribution using the stress of several discrete test points was proposed. To verify the performance of the developed test system and the validity of the proposed method, experiment was implemented using structural steel (Q235) specimens. Standard curves of sensors at each test point were achieved, the calibrated data were used to establish the BP neural network model for approximating the stress variation on the specimen surface, and the stress distribution curve of the specimen was obtained by interpolating with the established model. The results show that there is a good linear relationship between the change of signal modulus and the stress in most elastic range of the specimen, and the established system can detect the change in stress with a theoretical average sensitivity of -0.4228 mV/MPa. The obtained stress distribution curve is well consonant with the theoretical analysis result. At last, possible causes and improving methods of problems appeared in the results were discussed. This research has important significance for reducing the cost of eddy current stress measurement system, and advancing the engineering application of eddy current stress testing.

## Introduction

Ferromagnetic metal components are widely used in mechanical equipment. The measurement of stress and the estimation of stress distribution in them is the prerequisite for accurately obtaining the working status of components and mechanical equipment, and predicting their remaining service lifetime.

To evaluate the stress distribution on the surface of components, the stress should be measured at first. At present, the main nondestructive testing methods of stress include X ray diffraction, neutron diffraction, ultrasonic techniques and magnetic techniques [1–10], etc. Among these methods, magnetic techniques have the advantages of safety, low cost, highly sensitive and no need of coupling agent. In various magnetic techniques, eddy current stress measurement technology, based on piezoresistive effect and magneto mechanical effect, has attracted great attention of researchers, and has become one of the frontiers and focuses in the nondestructive testing of stress [11–14]. In recent years, researchers have done a lot of work in the mechanism and methods in eddy current stress measurement [15–23], which made great contribution to the research of eddy current stress measurement. However, only few researches in the testing of stress in ferromagnetic material have been reported. In addition, because a relatively high frequency signal, sometimes up to tens of MHz, is usually required for eddy current stress testing, ordinary electric measuring instruments cannot meet the requirements, many very expensive commercial instruments, such as impedance analyzer, high frequency digital multimeter and so on, are often used in current research. This leads to the high cost of eddy current stress measurement system, which greatly limits the application of eddy current stress measurement technology. Therefore, to establish a low cost eddy current stress measurement system to measure the stress in ferromagnetic specimens is an urgent problem to be solved in the research.

In evaluating the stress distribution on the surface of components, sensor array method and scanning method, are methods easy to be think of. The former obtains the stress of each point in the array by arrayed sensors, and indicate the stress distribution using the stress of these points. The latter obtain the stress of each point by scanning the sensor along the measured area, and then the stress distribution is obtained. But in some cases, these two methods are not available, for example, in the structural health monitoring, or part of the specimen is covered. In these cases, only the stress at several points on the specimen surface can be measured by eddy current testing. How to obtain the stress distribution on the whole surface using the stress of these measured points is a problem that needs to be solved.

In order to solve the above two problems, a low cost eddy current stress measurement system was established to measure the stress in ferromagnetic material, and a method based on BP neural network model for obtaining the stress distribution on the surface of the specimen was presented. Tensile experiment was implemented using structural steel specimens (Q235, in Chinese National Standard GB/T700-2006.). The validity of the established eddy current stress measurement system was verified, and the stress distribution curve on the surface of the specimen was obtained. At last, the possible causes of some phenomena appeared in the results were discussed in detail, and the directions of future work was also introduced.

## Materials and methods

### Materials

The designed planar eddy current sensor was fabricated in Jiangnan Computing Technology Research Institute (Wuxi, China). The tensile testing machine, with a maximum tensile capacity of 8 tons, was developed by Beijing Institute of Technology (Beijing, China). The designed test specimens were customized in Beijing Jinyu Electromechanical Equipment Co., Ltd. (Beijing, China), with the thickness of 4 mm, and all treated by stress relief annealing (preserved at 600±10°C for 2 hours, and then cooled in the furnace to room temperature). The material of the specimen is Q235, with the yield limit of 235 MPa. Signal generator, DC power supply, and digital oscilloscope were purchased from Beijing RIGOL Co., Ltd. (Beijing, China). The performance of the signal generator is as follows: the highest output frequency is 25 MHz, the sampling rate is 100 MSa/s, and the number of channels is 2. The DC power supply can output signals of ±25V/1A and +6V/5A. The performance of digital oscilloscope is as follows: the analog bandwidth is 100 MHz, the analog channel number is 2, the highest real-time sampling rate is 1 GSa/s, the maximum storage depth is 1 Mpts, and the digital channel is 16. The power amplifier was purchased from the Newtons4th (Leicester, UK), with performance as follows: can amplify signals from DC to 1 MHz, can produce a maximum output of ±20V peak/3A RMS and ±40V peak/3A RMS respectively at 1MHz and 500kHz. The designed board of signal conditioning circuit was fabricated in Shenzhen J&C Co. Ltd. (Shenzhen, China). The signal acquisition card is purchased from Beijing Youcai measuring and Control Technology Co., Ltd. (Beijing, China), with performance as follows: The resolution of 16 bits, 16/32 channel, and the highest sampling frequency of 250 kHz.

### Principle of eddy current technology for measuring stress

When a coil with alternating current is closing to a conductor specimen, the alternating magnetic field produced by the coil will induce a closed current in the conductor. The closed current is called eddy current. The magnetic field produced by the eddy current acts on the coil (or sensing coil), and result in changing in the impedance of the coil. The amplitude, phase and flow pattern of eddy current are influenced not only by the exciting coil and the exciting signal, but also by the specimen characteristics (conductivity, permeability, etc.) and the specimen status (stress, cracks, etc.). Therefore, the characteristics and status information of the specimen can be obtained by measuring the impedance change of the coil. This is the theoretical basis for that eddy current technology has been widely used in nondestructive testing and evaluation [24, 25].

Researches show that the conductivity and permeability of the metal specimen will change under the residual stress or applied stress. In non-ferromagnetic materials, the change of permeability is very small and can be neglected. At this point the main consideration is the change of conductivity caused by the piezoresistive effect. In the elastic range, the change of conductivity of the non-ferromagnetic material has an approximately linear relationship with the variation of the stress. Thus, the stress of the specimen can be measured by eddy current technique. In ferromagnetic materials, the situation is more complicated, because the permeability of the specimen also varies significantly with the change of stress. The reason for the variation of magnetic parameters of ferromagnetic materials with the applied stress is complicated, the nature of which belongs to the magneto mechanical effect. Researchers have proposed many different theoretical hypotheses [26–29], but still no theory has been able to explain all problems of the magneto mechanical effect. However, reported experiments show that, in the elastic range, there is an approximate linear relationship between the impedance change of the eddy current sensing coil and the stress at the detected area, therefore, the residual stress or applied stress of ferromagnetic specimens can also be measured by eddy current technique.

### Set up of eddy current stress measurement system

#### Scheme of eddy current stress measurement system

**Fig 1:**
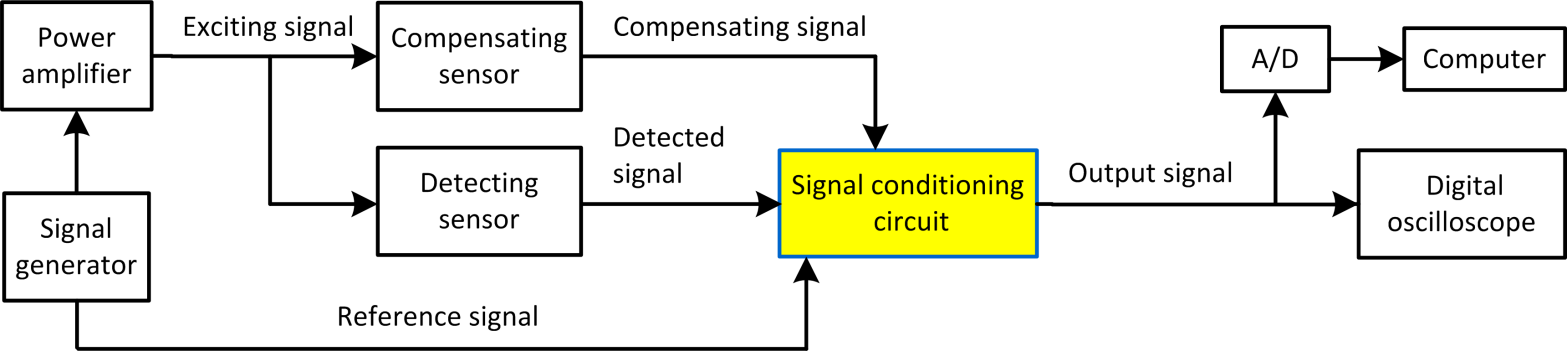
Schematic diagram of the eddy current stress measurement system.

**Fig 2:**
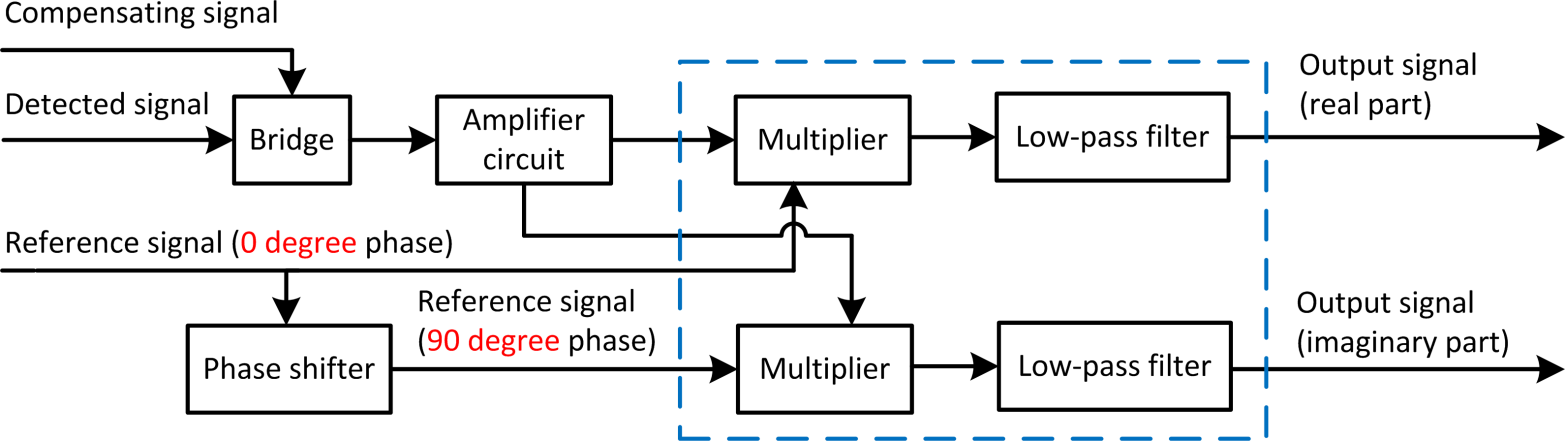
Block diagram of the signal conditioning circuit.

The schematic diagram of the eddy current stress measurement system is shown in Fig 1. A power amplifier is used to amplify the signal generated by the signal generator, to enhance the ability of the sensor in inducing the eddy current. Five detecting sensors and a compensating sensor are used in the system, but at the one time, only one detecting sensor is connected into the bridge in the signal conditioning circuit, as shown in Fig 2. The compensating sensor, with the same structure as the detecting sensor, is placed on a specimen without applied stress. The detecting sensor and the compensating sensor form the two adjacent arms of the bridge. Due to characteristics of bridge, outputs of compensating sensor and detecting sensor will be differenced. Thus the influence of change in ambient temperature can be compensated, the effects of space electromagnetic fields can be removed, and only the signal variation caused by the stress can be obtained. Thereby, the resolution of the measurement system is improved and the limitation for maximum input voltage of the subsequent circuit is reduced.

The block diagram of the signal conditioning circuit in Fig 1 is shown in Fig 2. The main functional modules include bridge, amplifier circuit and orthogonal lock-in amplifier. The pre balanced bridge outputs the modulated signal, with a frequency equal to the exciting frequency. The amplitude and phase of the modulated signal varying with the impedance change of the sensing coil. Due to the small amplitude of the modulated signal, amplifier circuit is needed to drive the subsequent circuit effectively. The amplifier circuit is composed of a two stage amplifier circuit and a low-pass filter, and these circuits are all based on ADA4817 amplifier. The first stage of the circuit is a differential amplifier, the second stage is a noninverting amplifier, and the low-pass filter, with amplitude amplification ability, is used to filter out the noise introduced by the former circuits. The orthogonal locked-in amplifier consists of the phase sensitive detector implemented by the multiplier (Based on AD835), and the low-pass filter (Based on AD826), as shown in the dashed frame in Fig 2, and finally outputs the real part and imaginary part signals that reflect the impedance change. These two signals are inputted, displayed and recorded in the digital oscilloscope or in the computer through the signal acquisition card, as shown in Fig 1.

The amplifier ADA4817 used in the signal conditioning circuit is a unity-gain stable, ultrahigh speed, voltage feedback FastFET amplifier with 1050 MHz −3 dB bandwidth developed with the Analog Devices, Inc., which can achieve ultralow noise as well as very high input impedances. The multiplier AD835 is a complete four-quadrant, voltage output analog multiplier also developed with the Analog Devices, Inc., which generates the linear product of its two voltage inputs with a −3 dB output bandwidth of 250 MHz. Therefore, the developed signal conditioning circuit has a wide bandwidth, which can meet the needs of using high frequency exciting signals in eddy current stress testing.

#### Principle of orthogonal locked-in amplifier in signal conditioning circuit

Assume that the frequency of the sinusoidal exciting signal is f, the initial phase is **θ**. According to the principle of bridge, the output signal of the bridge shall be in the form of **x**(**t**) as shown in Eq (1).

x(t)=Asin(2πft+θ+φ) (1)

Where, A is the amplitude of the signal, which affected by the detected signal and the bridge parameters; and φ is the phase change caused by unbalanced bridge when the impedance of the sensing coil changes with the stress in the specimen.

The unit reference signal with an initial phase of 0 degree can be expressed in the form of y(t) in Eq (2).

y(t)=sin(2πft) (2)

After multiplying by the multiplier, the signal becomes the form of u(t) shown in Eq (3).

$$u(t)=x(t)∙y(t)=\frac{A}{2}cos(\theta +\varphi )-\frac{A}{2}cos(4\pi ft+\theta +\varphi )$$ (3)

After filtering by the low-pass filter, the high frequency component of frequency at 2f will be filtered out, and the signal is changed to the form of U_x_ as shown in Eq (4). Obviously, U_x_ only changes with the impedance of the sensing coil caused by the stress change.

$$U_{x}=\frac{A}{2}cos(\theta +\varphi )$$ (4)

The unit reference signal with an initial phase of 0 degree is inputted into a phase shifter in Fig 2, and the initial phase of which is changed to 90 degree. After multiplying with x(t) and low-pass filtering, we obtain *U*_*y*_ as shown in Eq (5).

$$U_{y}=\frac{A}{2}sin(\theta +\varphi )$$ (5)

*U*_*x*_ and *U*_*y*_ are a pair of orthogonal signals, which respectively reflect the real part and imaginary part of the impedance change. It can be seen that the established system can obtain the impedance change of the sensing coil, without using impedance analyzer. Thus, the cost of establishing the eddy current stress measurement system is reduced.

#### Low cost measurement of exciting current

**Fig 3:**
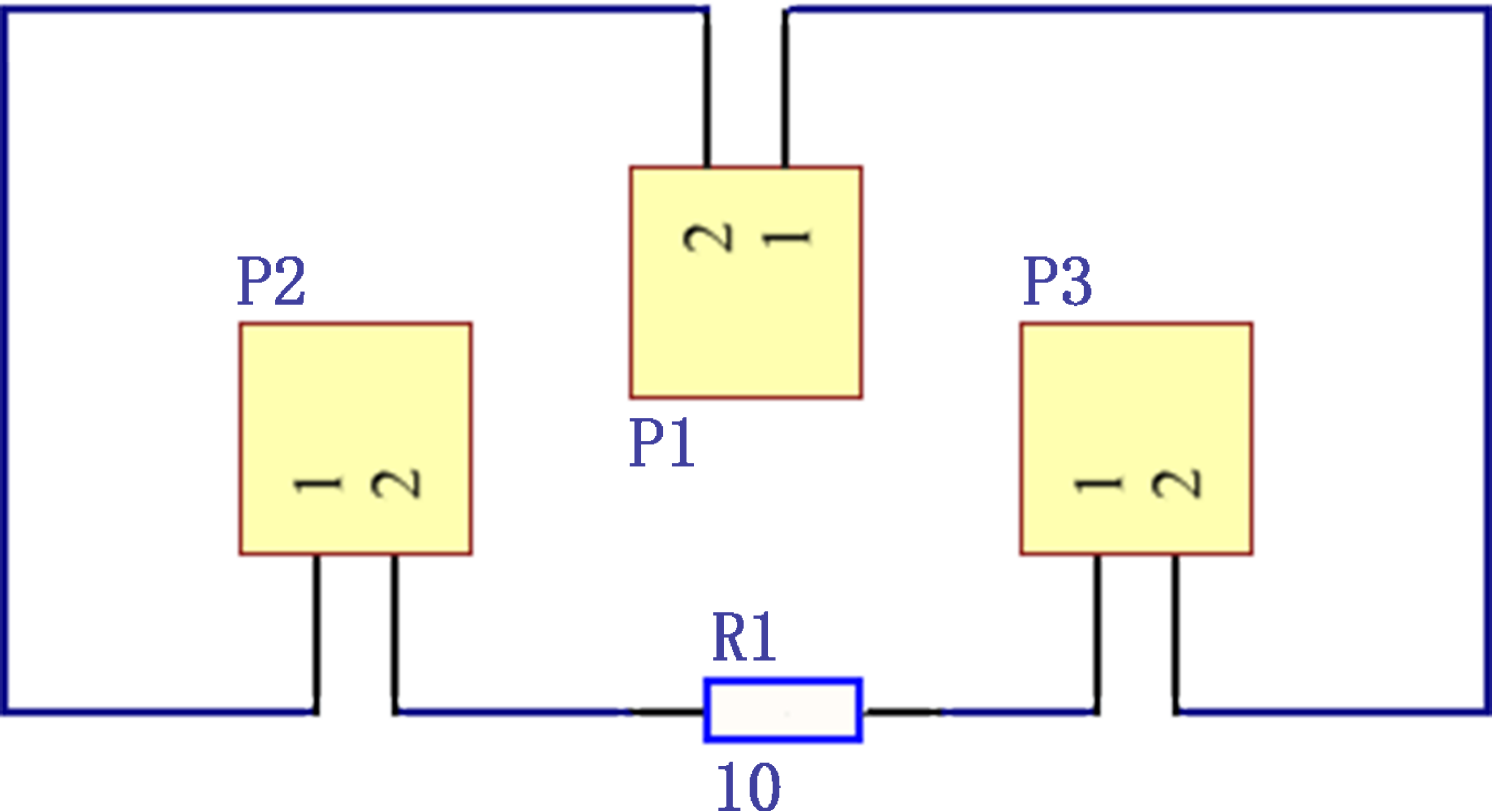
The current measurement scheme.

In analyzing the detected signal, it is necessary to measure the exciting current intensity. In general, current is measured using a multimeter, but in the measurement of stress using eddy current method, due to the possible high frequency of exciting signal, the ordinary multimeter cannot measure the current correctly, and commercial high frequency multimeters are always very expensive [30]. In order to reduce the cost of the measurement system, the current measurement scheme shown in Fig 3 was adopted, which was arranged in the signal conditioning circuit board, where P1 is the input terminal of the total exciting signal, P2 is the terminal for providing exciting signal to the sensing sensor, P3 is the terminal for providing exciting signal to the compensating sensor, and R1 in series is a 10 ohm high precision resistor with a maximum power of 5 W. The voltage across the R1 can be measured with an oscilloscope, and the exciting current in the loop can be calculated. Because oscilloscope with the bandwidth of tens of MHz is relatively common and cheaper, so this scheme can also reduce the cost of establishing eddy current stress measurement system.

### Design and simulation of the sensor

The traditional eddy current sensor is fabricated with winding wire around the ferrite, although it is easy to be made, and can output strong signal, but it also has many disadvantages, such as larger volume, larger inertia in dynamic scanning, and difficult to detect complex surface. In recent years, planar eddy current sensor fabricated by micromachining technology has attracted more and more attention of researchers [31–34]. Planar eddy current sensor is small in size and light in weight, can be used to make complex scheme, and easy to array. If it is made with flexible materials, it also can be adapted to detect various complex surfaces. More importantly, when mass-produced, the cost is very low and it is easy to ensure the consistency of the sensors. For these reasons, the planar eddy current sensor was adopted in this study.

**Fig 4:**
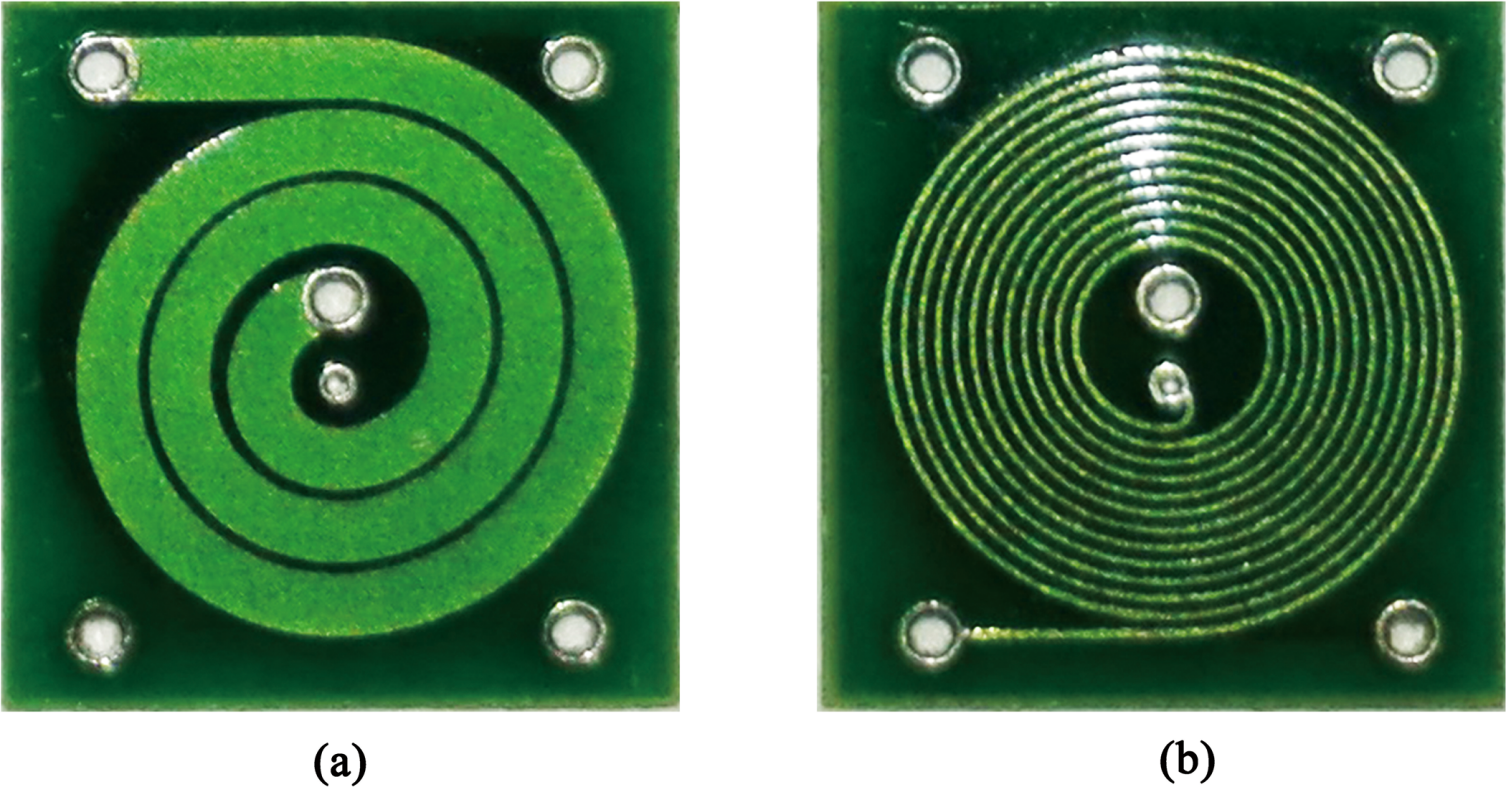
Appearance of the sensor.

In order to use strong exciting current, the exciting coil and the sensing coil are respectively configured in different layers of the circuit board. To obtain relatively strong detected signal in the limited detected area, four-layer circuit board was used to fabricate the sensor. Exciting coils were arranged in top two layers in series through via, and sensing coils were arranged in bottom two layers in series also through via. In various plane curves, the Archimedes spiral is the closest curve to traditional coil in the structure, which can be considered as the variation of traditional sensor compressed to the plane along its axial. So we chose the Archimedes spiral as the curve of the coil. The top surface and the bottom surface of the fabricated sensor are shown as Fig 4(A) and 4(B), respectively. Pads at four corners are used to weld the wire. In Fig 4(A), the two pads on the left are used to input the exciting signal, and the two on the right are used to output the detected signal. Exciting coil circles 3 laps in every layer with an interval of 0.1016 mm between each lap, and the line width of the coil is 0.7620 mm. Sensing coil circles 12 laps in every layer with an interval of 0.1016 mm between each lap, and the line width of the coil is 0.1016 mm. Diameters of the exciting via and its hole respectively are 0.7620 mm and 0.4064 mm. Diameters of the sensing via and its hole respectively are 0.5080 mm and 0.2540 mm. The thickness of each layer in the sensor is about 0.1270 mm, and the overall size of the sensor is 7.6200 mm×7.6200 mm×0.5080 mm.

**Fig 5:**
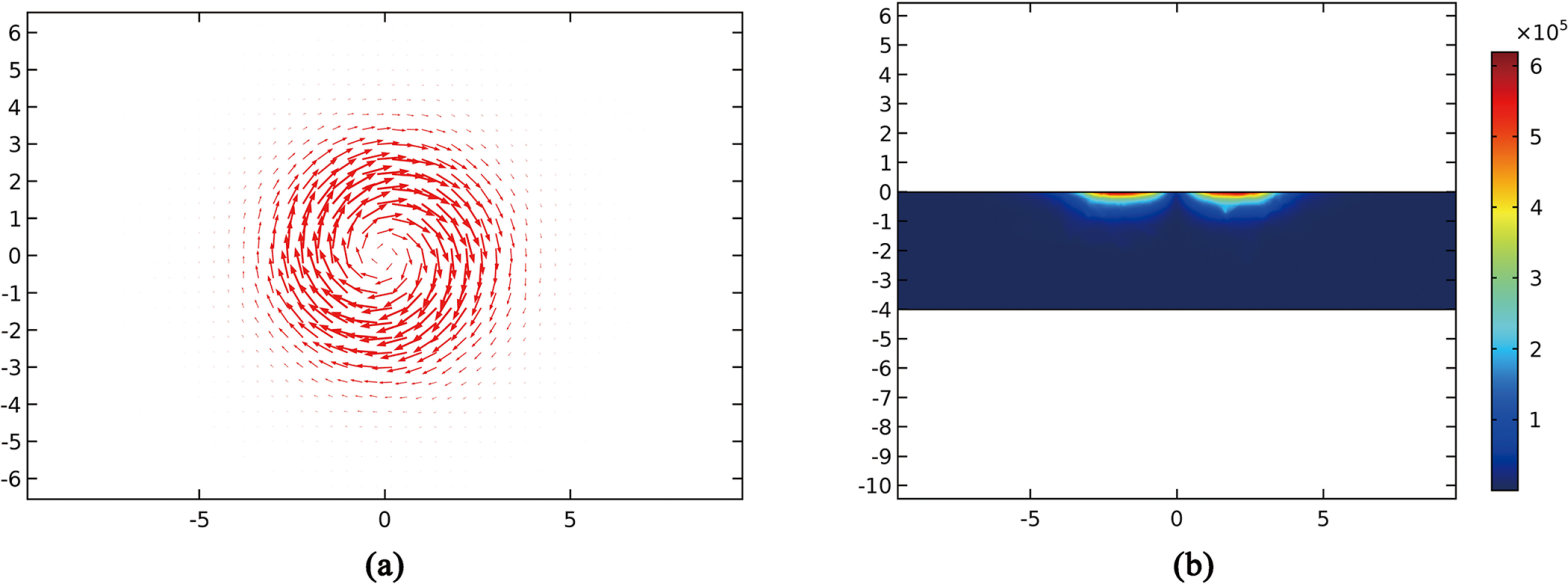
Simulation results of the sensor.

To preliminary verify the validity of inducing eddy current by the designed sensor, simulation analysis was carried out. The exciting coil simulation model configured at two layers in series was established in COMSOL Multiphysics, according to the above curves. The tested conductor material was selected as structural steel, whose size was set as 40 mm×40 mm×4 mm, and the simulated spatial area was set as 100 mm×100 mm×50 mm. The space medium was selected as air. Exciting coils in the lower layer and in the upper layer were respectively located at 0.310 mm and 0.437 mm above the center of the conductor (Approximately consistent with the situation of experiment.). Edge current was inputted into the coils. Sinusoidal signal of 500 kHz, with 0.1 A current intensity, was used as exciting signal in the simulation. The eddy current on the surface of the specimen induced by the sensor is shown in Fig 5(A), which validated that the sensor can effectively induce eddy current. Sliced along the center line of the top surface of the specimen, and obtained the current density modulus distribution in the cross section of specimen (Fig 5(B)). It can be seen that the eddy current was concentrated below the sensor and decreased along the thickness direction of the specimen.

### Method to estimate the stress distribution based on BP neural network

#### The necessity of using BP neural network to deal with the stress distribution

In the estimation of stress distribution on the surface of components, if sensor array and scanning method couldn’t be used and only stress at several discrete points on the surface of specimen could be measured using the eddy current technology, the only method is try to find the stress variation rule from the stress of the measured points, and then estimate the stress of other unmeasured points. In essence, this problem belongs to the function approximation and interpolation problem. In various methods of function approximation, polynomial fitting and cubic spline interpolation are two widely used methods. These two methods are simple and easy to use, but if the data changed complexly, the fitting accuracy is difficult to be guaranteed. Moreover, if only few data points are available, in high order polynomial fitting and cubic spline, there would be distortions at the two ends of the data, that is, the endpoint effect. The closest way to the method of analyzing data changes in the human brain is neural network technology, and it should be feasible to approximate the stress variation rule hidden in the stress data of measured test points using neural network model. In all kinds of neural networks, the error backpropagation neural network model (BP neural network) has been widely used in data fitting because of its good generalization ability [35]. Studies have shown that 3 layer BP neural network can theoretically approximate any continuous function and its derivatives at any precision [36]. Therefore, in this study, the BP neural network model was used to approximate the stress variation rule and estimate the stress distribution on the surface of specimen.

#### Preparation of training samples and test samples

BP neural network was used to approximate the stress variation with the position in this research. Therefore, the input data of the neural network are the spatial coordinates, and the output data are stress values at these positions. In order to reduce the difficulty of training the model, it is necessary to normalize the data because the values of coordinates and stress at each position are generally large. For the sake of simplicity, these data were normalized using the peak method. That is, the data to be normalized are divided by a value (normalization factor) slightly larger than the possible maximum value in these data, thus converting the input and output data into normalized data between 0~1. Normalization factors of input data and output data should been set separately.

The measured points are classified into training points (80%~90%) and test points (10%~20%). The normalized input and output data of training points are used as training samples, and the normalized input and output data of test points are used as test samples.

#### The procedure of establishing BP neural network model

To establish the BP neural network model, the structure and related parameters of BP neural network must be set firstly. After selecting appropriate training methods, training samples are used to train the model and test samples are used to test the model. When training errors and test errors are all within the allowable range, the training is finished, and the rule of stress variation in measured area has been achieved.

#### Method for obtaining stress distribution

The appropriate interpolation spatial interval should be set according to the needs of analyzing the stress distribution, and then the interpolation spatial coordinates array is produced. After normalization, the coordinates array is input into the trained BP neural network model; the data of output array generated by the model are the normalized stress values corresponding to the interpolation coordinates. To obtain the actual stress distribution, the output data of the model need to be treated by inverse normalization corresponding to the aforementioned normalization method. The output data should be multiplied with the same normalization factor as normalized, thus the stress at the interpolation spatial coordinates are obtained, and then the stress distribution curve or map can be achieved.

## Experiment and results

### Stretch experiment

**Fig 6:**
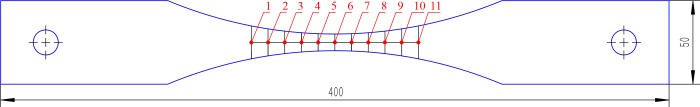
Tensile specimen.

The tensile specimen was designed as shown in Fig 6. The middle of the specimen was designed as tapered arc, to ensure the maximum stress located at the middle of the stretched specimen, and the stress varies along the direction of the central axis. Two holes at both ends were used to fix the specimen on the tensile testing machine. Drawn a line along the central axis of the specimen, took the center position of the drawn line as the starting point, drawn a transverse line perpendicular to the central axis every 10 mm forward both ends, marked each intersection point of each transverse line and the central axis, and numbered the marked points from test point 1 to test point 11 from left to right, as shown in Fig 6. The compensating specimen and specimen to be stretched were marked in the same way. Before the experiment, on the specimen to be stretched, sensors welded wires had been respectively carefully affixed using adhesive tape at test point 2, test point 4, test point 6, test point 8, and test point 10, and attentions had been paid to keep each mark at the center of each sensor. Since there was only one compensating sensor, in subsequent procedures, it was all ensured that the position of the compensating sensor on the compensating specimen was the same as the position of the detecting sensor currently accessed into measurement system on the stretched specimen.

**Fig 7:**
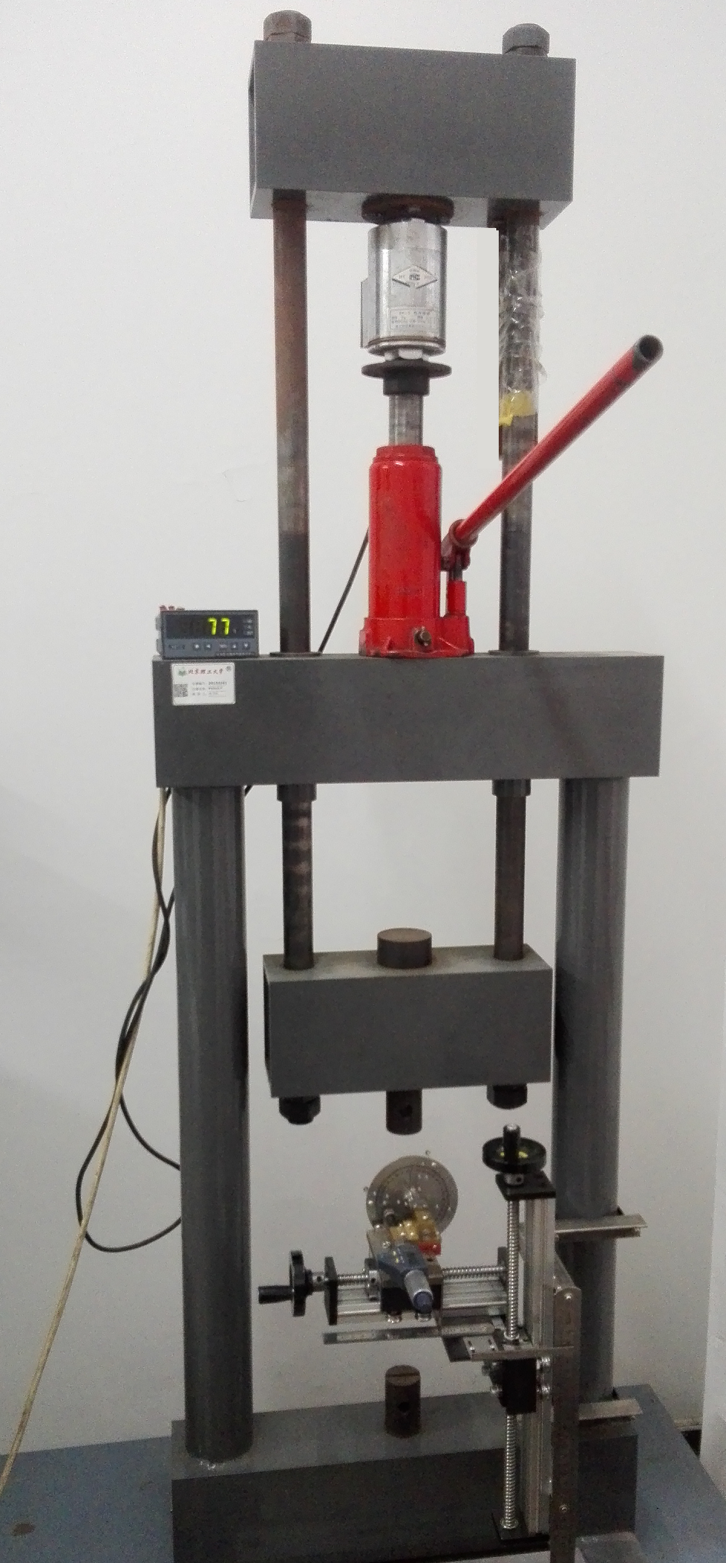
The tensile testing machine.

The tensile testing machine used in the experiment is shown in Fig 7. The red hydraulic jack can be operated by the red lever to realize the loading of the specimen. The value of tensile force can be measured by the force sensor and displayed in the display device. In the beginning of the experiment, adjusted the distance between the two clamping heads of the tensile testing machine to fix the specimen between the clamping heads using a bolt (Diameter: 15 mm.), and paid attention to ensure that there is no shaking and no pretension.

Before starting the stretching, the sensor at test point 6 (Fig 6) was connected into the circuit to adjust the circuit parameters. At first, the components in the bridge were adjusted to get the minimum amplitude of the output signal of the bridge excited with 500 kHz signal. Then the amplitude of the exciting signal was adjusted to ensure the real part signal and imaginary part signal were all in the proper range. After the adjustment, the peak of the exciting current intensity was 160.5 mA, measured by the method introduced in above.

Sensors at each test point were connected into the circuit in turn, and the test signals were input into the oscilloscope and the computer. Since this is a static stretch experiment, the ideal test signal of each test point should be a constant value when the tension is constant. To eliminate the influence of noise, respectively filtered the real part signal and the imaginary part signal using low-pass digital filters with bandwidth of 200 Hz, and then respectively calculated ${Re}_{i,j}^{n}$ and ${Im}_{i,j}^{n}$, where ${Re}_{i,j}^{n}$ and ${Im}_{i,j}^{n}$ are respectively the mean of 2 millisecond length filtered real part signal and imaginary part signal at the ith test point of the jth specimen under n kilogram-force tensile force. Recorded ${Re}_{i,j}^{0}$ and ${Im}_{i,j}^{0}$. Then the tensile force was gradually increased from zero, every 100 kilogram-force increased, ${Re}_{i,j}^{n}$ and ${Im}_{i,j}^{n}$ were recorded once, until the tensile force reached 900 kilogram-force. At that time, the stress in the middle of the specimen had already close to the limit of its elastic range. Replace another specimen and repeat the above procedures until 5 specimens all had been stretched.

Calculated ${Re}_{i}^{n}$, ${Im}_{i}^{n}$, ${Mod}_{i}^{n}$, and ${CSM}_{i}^{n}$, respectively as shown in equations from Eq (6)–Eq (9). ${CSM}_{i}^{n}$ is the change of signal modulus, in nature, it reflects the change of impedance in sensing coil.

$${Re}_{i}^{n}=\frac{1}{5}\sum_{j=1}^{5}{Re}_{i,j}^{n}$$ (6)

$${Im}_{i}^{n}=\frac{1}{5}\sum_{j=1}^{5}{Im}_{i,j}^{n}$$ (7)

$${Mod}_{i}^{n}=\sqrt{{\left({Re}_{i}^{n}\right)}^{2}+{\left({Im}_{i}^{n}\right)}^{2}}$$ (8)

$${CSM}_{i}^{n}={Mod}_{i}^{n}-{Mod}_{i}^{0}$$ (9)

### Establishment of the standard curve for the quantification of the stress

**Fig 8:**
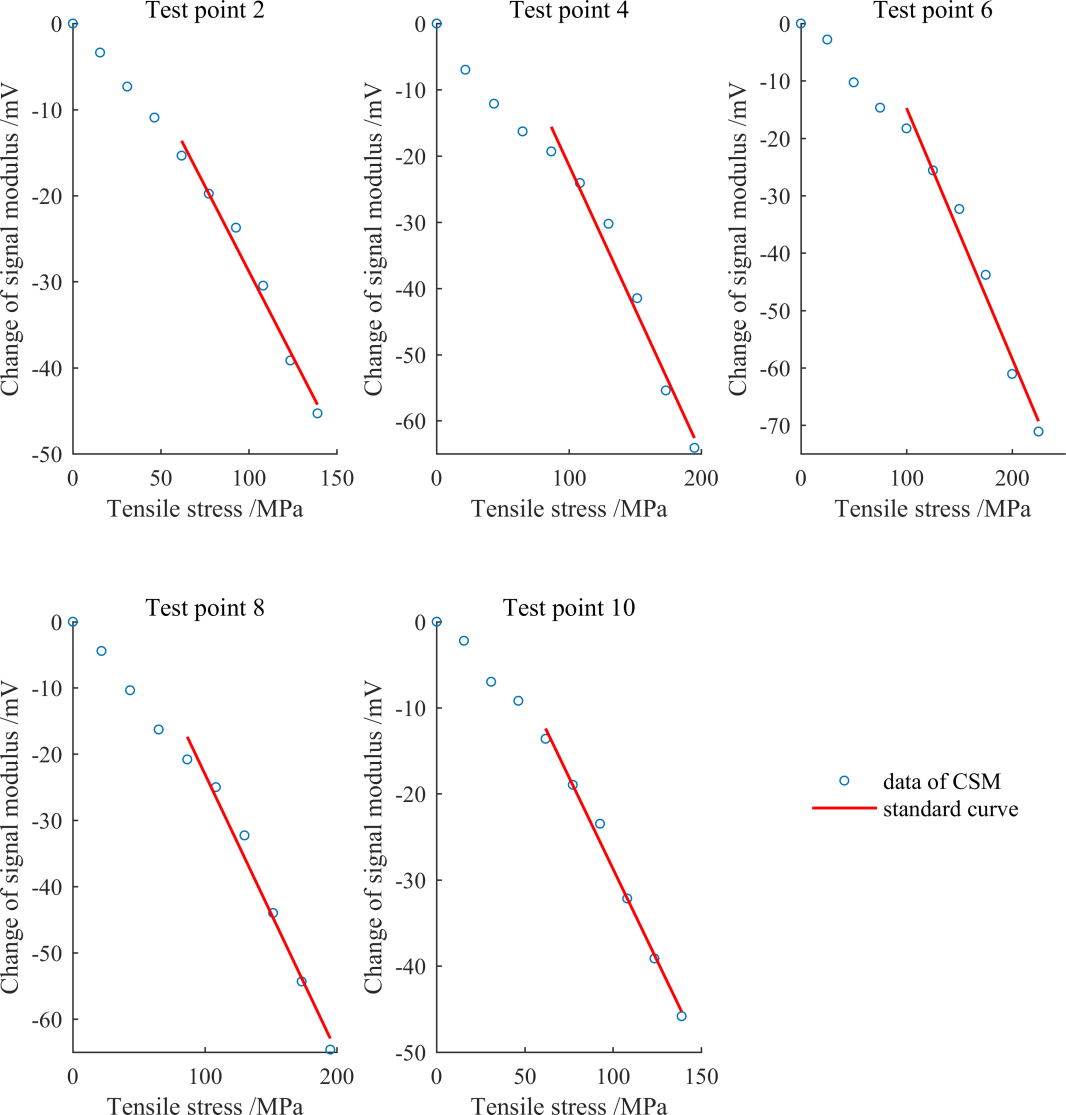
Changes of signal modulus and the calibrated standard curves.

Since the parameters of the arc and other geometric dimensions of the tensile specimen are known, the theoretical stress of each test point under each stretch can be calculated. The change of signal modulus of each test point was plotted as ordinate, and the corresponding stress was plotted as abscissa. The result is shown as the blue small circle in Fig 8. It can be seen that there is an obvious nonlinearity in the first three stretches. In order to obtain more practical standard curves, it was determined that all the calibration curves of every test point were established using the data start from the fourth stretching, based on least square method. The established stress calibration curves of each test point are shown as red solid lines in Fig 8. The calibration equations and values of R^2^ of each test point are shown in Table 1, where CSM is the change of signal modulus, and σ is the stress. The theoretical average sensitivity of five test points is -0.4228 mV/MPa. Obviously, there is a linear relationship between the stress and the change of signal modulus. Therefore, it is feasible to measure the stress of ferromagnetic specimens using the established low cost eddy current stress measurement system.

**Table 1:** The calibration equations and values of R^2^ of each test point.

ID of test point	Calibration equation	R^2^
2	CSM = -0.3970×σ3 10.8953	0.9819
4	CSM = -0.4340×σ4 22.0080	0.9721
6	CSM = -0.4365×σ4 28.9214	0.9745
8	CSM = -0.4206×σ419.0610	0.9806
10	CSM = -0.4261×σ4 13.9211	0.9918

### Establishment of BP neural network interpolation model

**Fig 9:**
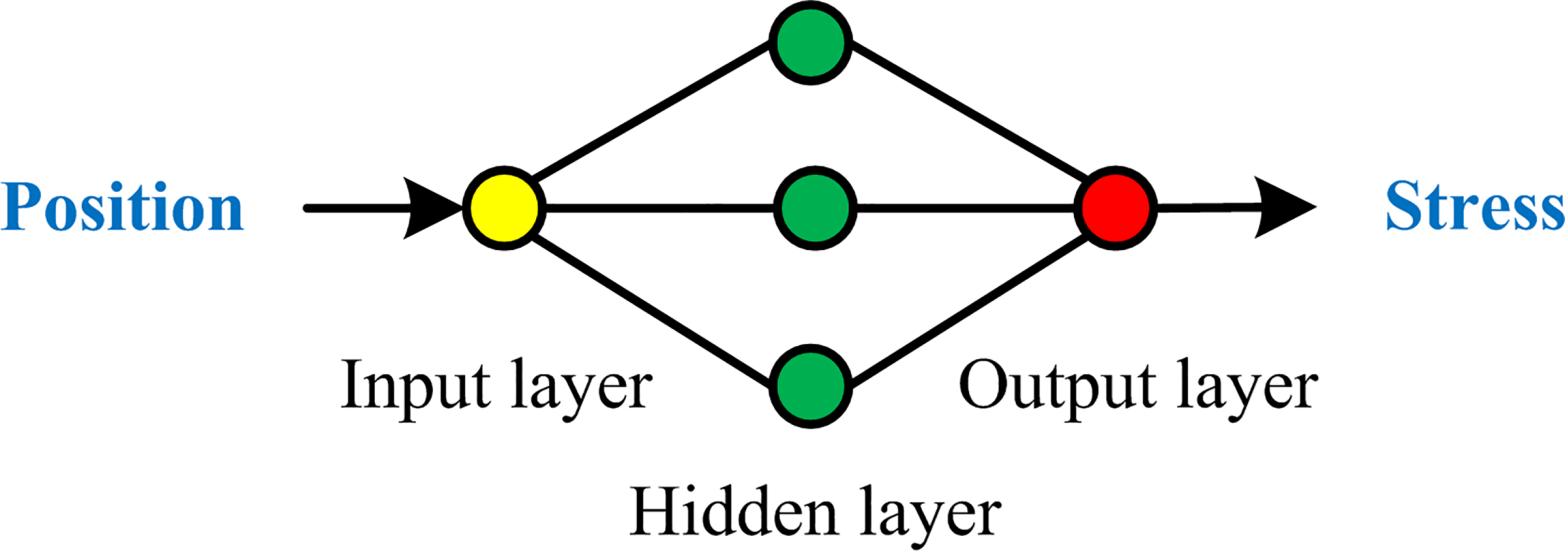
The structure of BP neural network model.

The number of neurons in the input layer was determined as 1, to input the space coordinates. The number of hidden layer was determined as 1. The number of neurons in the hidden layer was determined as 3, based on the result of several experiments. The number of neurons in the output layer was determined as 1, which was used to output the corresponding stress. Tangent and logarithmic sigmoid functions were adopted as activation functions of neurons in hidden layer and output layer, respectively. The structure of the neural network model is shown in Fig 9. In this study, the reason for there was only one neuron in the input layer is that the inputted coordinates is only the locations of test points at the central axis of specimen. If multidimensional coordinates were needed, the number of neurons in input layer should also be increased.

Took test point 1 as the origin point, the direction to test point 11 as the positive direction, to determine the spatial position of each test point (The unit is mm.). According to the actual situation, took 100 mm as the input normalization factor to normalize the spatial position, and took the normalized position data of point 2, point 4, point 6, point 8 and point 10 as input training samples. Took the calibrated stress of corresponding test point under a certain tensile force as the output data to be normalized (In this article, took the data under the tensile force of 600 kilogram-forces as an example.). Took 250 MPa as the output normalization factor to normalize the output data, and took the normalized output data as output training samples. In order to obtain the test sample, during experiment, under 600 kilogram-forces, the sensor at test point 10 was moved to test point 3 to detect the stress of test point 3, and then was pasted back to the position of test point 10. The position of test point 3 and the stress calibrated by the calibration curve of test point 10 were normalized in the same way as used in the training data to respectively obtain the input test sample and output test sample.To shorten the training time and avoid the local convergence, the Levenberg-Marquardt optimization algorithm was used to train the neural network [37]. The goal of training is the mean square error less than 0.0001. After repeated training, the goal was achieved.

### Estimation of the stress distribution on specimen

**Fig 10:**
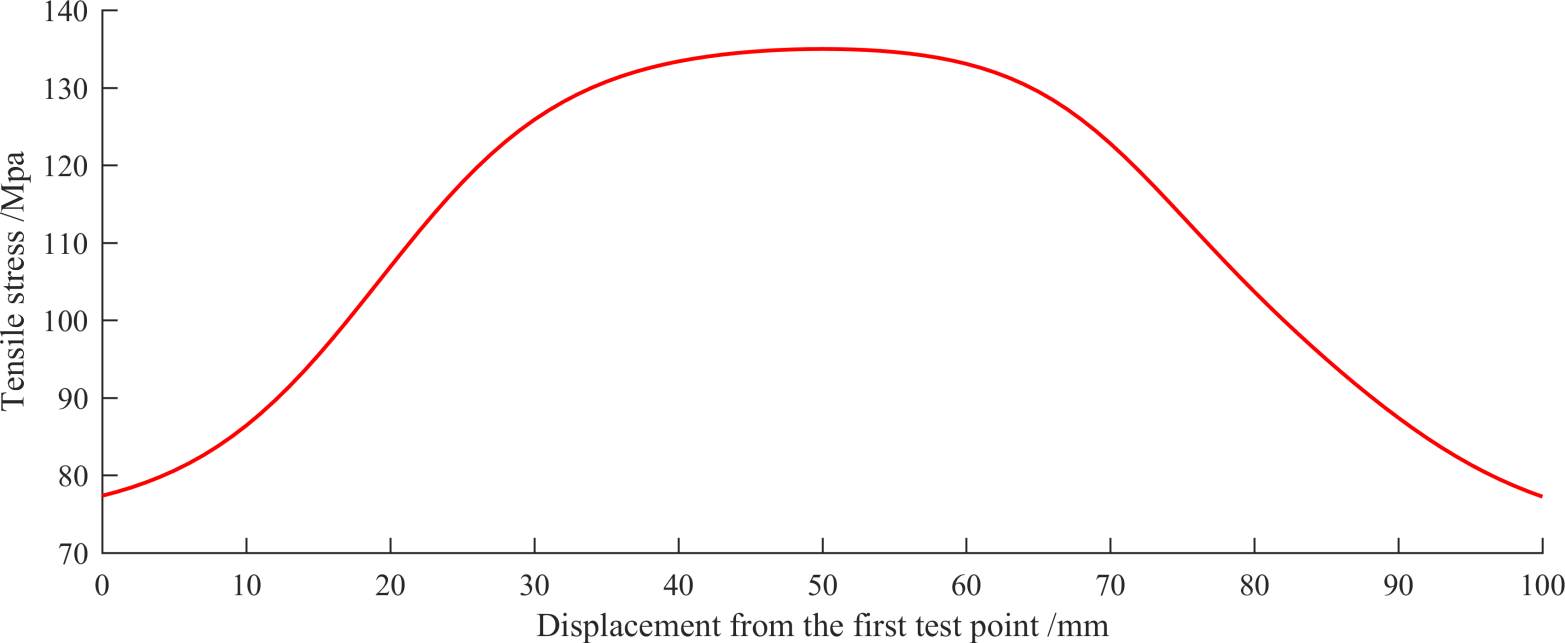
Stress distribution curve in the middle area of the central axis on specimen (under the tensile force of 600 kilogram-forces).

In order to obtain the stress distribution on the surface of the specimen, the input vector used for interpolation must be generated firstly. From 0 mm to 100 mm, every 1 mm, generated a position as an element of the input vector, thus the input vector was obtained. The input vector normalized by the above method was inputted into the trained BP neural network model, and the output vector was the corresponding interpolated stress need to be inverse normalized. Fig 10 shows the stress distribution curve obtained by interpolation. It can be seen that the maximum stress located at the middle position of the specimen, the minimum stress located at the coordinates of 0 mm and 100 mm, and the stress distribution curve is symmetrical about 50 mm. The result is consistent with the actual stress variation of the specimen, which validate that the proposed method can estimate the stress distribution using stress data of several test points.

## Discussion

### Cost analysis of the established measurement system

To demonstrate the low cost of established eddy current stress measurement system, the cost of this system was compared with that of the system using wide bandwidth commercial instruments. Because the signal generator, the DC power supply, the power amplifier, and the acquisition card are widely used in various eddy current stress measurement systems, the cost of these modules were not included in the following comparison.

The cost of the signal conditioning circuit board and sensors used in this system is not more than $40, and the price of the 100MHz bandwidth digital oscilloscope used in this system is less than $900. While the impedance analyzer with more than 10MHz bandwidth is generally priced above 10 thousand dollars, and the digital multimeter with 10MHz bandwidth is sold for thousands of dollars.

The cost of the established measurement system is only less than 10% of the cost of system using wide bandwidth commercial instruments. Obviously, the established eddy current stress measurement system can measure the stress at a low cost.

### Characteristic analysis of the stress measurement system

#### Causes of the low sensitivity

It can be seen from the results, though there is a good linear relationship between the stress and the change of signal modulus, the sensitivity of the sensor and eddy current stress measurement system is low. Possible causes for this phenomenon are discussed as follows.

- Bridge had not been completely balanced

In the experiment, it is difficult to adjust the bridge to completely balanced, therefore, in practice, the signal outputted by the bridge became minimum, not the ideal zero, was used as the criterion of balance. This made the bridge still output signal with certain amplitude even if the specimen is not subjected to tension. After further amplification, the amplitude is further increased; result in a larger initial value of the final output. To avoid the amplifier circuit to be saturated, the maximum exciting current through the sensor was limited. And to avoid the amplified signal beyond the input range of the multiplier in orthogonal locked-in amplifier, the maximum magnification of amplifier circuit was also limited. Thus the sensitivity was limited in a low level.

- Design of the sensor needs to be improved

**Fig 11:**
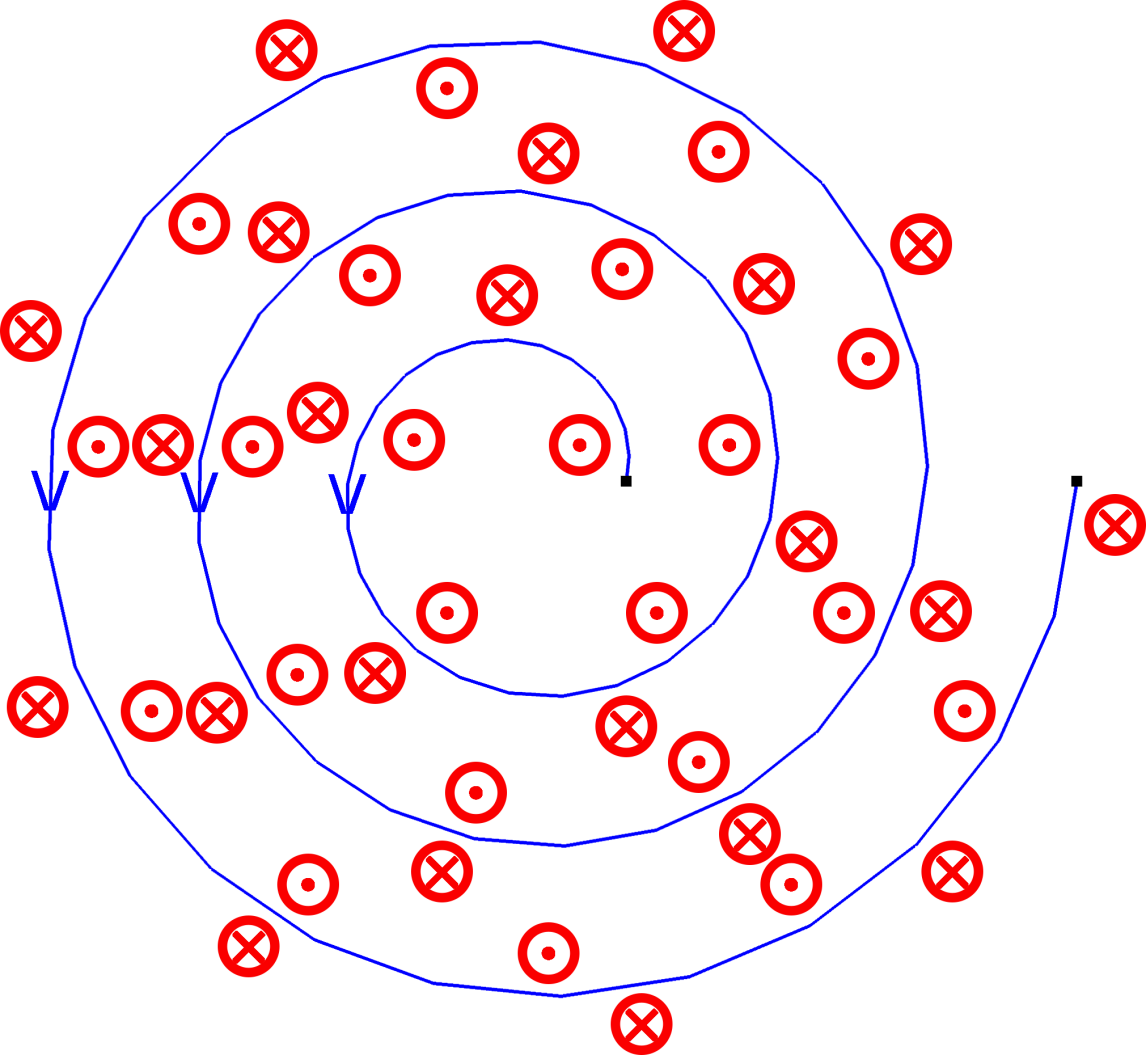
Directions of magnetic field produced by Archimedes spiral coil.

Although the designed Archimedes spiral exciting coil can induce eddy current on the specimen surface, as the simulation results shown, the eddy current is concentrated below the exciting coil, but in this area, directions of magnetic field produced by two adjacent circles are opposed, which counteracts part of the change in magnetic field (Fig 11). This reduces the induced eddy current intensity and affects the sensitivity.

According to the above analysis, we can start from the following aspects to improve the sensitivity of the system: (1) Try to implement an automatically balanced bridge to completely eliminate the compensating signal. (2) Try to design novel sensors to reduce mutual counteraction of magnetic fields between adjacent circles.

#### Causes of differences in sensitivity consistency

Each detecting sensor was calibrated in the experiment. Although the calibration curves are close to each other, there are differences both in the curve slope and the intercept (Table 1). This shows that the sensitivity consistency of sensors at different test points is not very good. Possible causes for this phenomenon are discussed as follows.

- Fabrication accuracy of the sensor is inconsistent

Due to the small size of sensors, the small differences between the sensors in fabrication will lead to changes in sensor performance, resulting in inconsistent sensitivity.

- Spatial variation of metallographic structure in specimen

The metallographic structure in the specimen cannot be exactly the same in all areas, and the accidental differences in microstructure are naturally reflected in the consistency of sensitivity. Since it is impossible to get the exact same specimens, the influence in this respect cannot be completely eliminated.

- Micro changes in sensor lift off due to the fixed method

In the experiment, the sensor was pasted with tape along the direction perpendicular to the axis of the specimen. For the gradual change of the specimen size, the difficulty of sticking at different test point is different. Although already pasted as carefully as possible, the micro distance between the sensor and the specimen might be inconsistent. This leads to differences in micro lift off, which in turn affects the sensitivity consistency.

- Limitation of the experiment method and the equipment

In the experiment, only one test point was accessed into the signal conditioning circuit at once time, a short period of time was consumed in switching sensors in turn, under the same stretch. The tension of the tensile testing machine used in the experiment was maintained by the hydraulic jack, during the consumed time, actual tension maybe had already changed due to the possible minor unloading of the hydraulic cylinder. This may also affects the consistency of sensitivity.

According to the above analysis, the possible methods to improve the sensitivity consistency including: (1) Fabricate more accurate sensors. (2) Improve the fixing method of sensors, and increase the uniformity of initial micro lift off. (3) Design multi-channel signal conditioning circuit to shorten the test time, or purchase new stretching equipment.

#### Causes of nonlinear characteristics at low stress

As shown in Fig 8, in the first three stretches, the change of signal modulus of each test point presented obvious nonlinear characteristics, which limits the use of the sensor and measurement system in stress measurement. Possible causes for this phenomenon are discussed as follows.

- Micro changes in sensor lift off due to the tightening of the tape

When the tensile force was small, the tape was only slightly taut, with the increase of tension, the tape was also tightened. This makes the micro lift off between the sensor and the specimen decreased, thus changed the change of signal modulus. When the tensile force increased to a certain extent, the lift off had been too small, almost not changed with the increase of tension, and the change of the change of signal modulus became stable. This explanation is consistent with the changes shown in Fig 8.

- Internal stress of specimen

Although the specimen had been subjected to stress relief annealing, new small internal stress may be introduced during transportation and handling. When the tensile force was small, this effect might not be neglected. But in practical, there is no way to avoid the internal stress of the specimen entirely. What we can do is only try to reduce its influence on the measurement results.

- Characteristics of ferromagnetic materials

Even if not been stretched, the magnetic domains of ferromagnetic materials are still subjected to the effects of geomagnetic and environmental magnetic fields. Although this effect is minimal, it may not be neglected when the tension was small.

According to the above analysis, further research will be done to reduce the nonlinear characteristics at low stress: (1) Improve the fixing method to reduce the change of micro lift off with the load; (2) Apply magnetic saturation or demagnetization to the specimen, to reduce the influence of ferromagnetic characteristics.

#### Causes of differences in correlations

As shown in Table 1, the correlations between the stress and the change of signal modulus at each test point are different. R^2^ of test point 6 and test point 4 are relatively small, and the closer to two ends, R^2^ become larger. This should owe to the edge effect of the eddy current. Test point 6 and test point 4 are all close to the middle of the specimen, the width of the middle of the specimen is only 10 mm, but the width of the sensor is 7.62 mm, the edge effect is significant, result in the small correlation. The closer to the end, the width of the specimen becomes wider, the edge effect becomes smaller, and correlation becomes higher. The change of R^2^ basically agrees with this.

In order to avoid this phenomenon, attention should be paid to ensure that the distance from the test point to the edge of the specimen is much greater than the sensor size.

As compared with the R^2^ of the test point 8 and test point 10, those of test point 2 and test point 4 are a little small, probably because of the asymmetry of the microstructure in the compensating specimen.

It needs to be pointed out that the noise, the recording error and the calculation error may also affect the above discussed contents.

### Analysis of interpolation accuracy of stress distribution

The main factors that affect the interpolation accuracy are measurement accuracy and model accuracy. Between them, the influence of measurement accuracy is the root cause. If the measurement accuracy is low, it will inevitably lead to the distortion of the stress distribution obtained by interpolation. If the measurement accuracy can meet the requirements, model accuracy is usually easy to meet the requirements. As long as the model structure is reasonable and the number of test points is enough, in general, the proper stress distribution can be obtained by this method.

Therefore, the premise of obtaining accurate stress distribution is to measure the stress in high accuracy.

In addition, it is important to be pointed out that: there were only 5 sets of data were used to train the BP neural network in this experiment due to the simple variation rule of stress and the limited sizes of the designed sensors and specimens, but it may cause an error for the same type problem in other study using less training data. In fact, in training the BP neural network, we should use as much training data as possible to improve the precision of approaching the stress variation rule and avoid the generalization error.

## Conclusions

The precise knowledges of stress and its distribution on the surface of ferromagnetic metal components is the key to the status analysis and life prediction of the equipment. The method of measuring the stress using eddy current technique is a promising pathway in this field. However, only little researches have been done on measuring the stress of ferromagnetic specimens using this method, and costs of currently established eddy current stress measurement systems are usually very expensive. To measure the stress by eddy current technology at low cost, a low cost measurement system was established, and in which a planar eddy current sensor based on Archimedes spiral was presented. The experiment using Q235 structural steel verified the validity of the established measurement system in the stress measurement of ferromagnetic specimens. The establishment of this system was a useful attempt to reduce the cost of eddy current stress measurement system. It is significant to reduce the funding threshold in the research of eddy current stress measurement. It is significant to further develop portable low cost eddy current stress testing devices. And it is significant to promote the engineering applications of this technology.

In order to estimate the stress distribution on the specimen surface without scanning, a method based on interpolation using BP neural network model was proposed for the first time. The interpolation results verified the feasibility of the method. This method has broad application prospects. It can be used to obtain the stress distribution on two-dimensional or three-dimensional object surface, and then find out the stress concentration area. It also can be used to evaluate the residual stress distribution on the surface of the components.

Regarding some problems indicated in the experimental results, the possible causes were analyzed, and directions of further research were also introduced. It is valuable for developing low cost and high performance eddy current stress measurement system and establishing more accurate stress distribution model in the future.

In the following work, we will develop an automatically balanced circuit to completely remove the compensating signal, increase the number of channels in the signal conditioning circuit, design more novel sensors, and improve the experiment and the fixing method of sensor, to improve the sensitivity, accuracy and reliability of the stress measurement system and achieve more accurate stress distribution.

## Supporting information

S1 File Parameters of the established neural network. (DOCX)
