# Supplementary material for: Estimation of stress distribution in ferromagnetic tensile specimens using low cost eddy current stress measurement system and BP neural network
Source: PLoS One. 2017 Nov 16;12(11):e0188197. doi: 10.1371/journal.pone.0188197 (PMC5690613; doi:10.1371/journal.pone.0188197)
Supplement: S1 File — (DOCX) [file pone.0188197.s001.docx]

**The weights of connection between the input layer and the hidden layer**

| **ID of neurons in hidden layer** | **1** | **2** | **3** |
| --- | --- | --- | --- |
| **Weight of connection** | 9.5127 | -7.1243 | 7.3938 |

**Threshold of neuron in hidden layer**

| **ID of neurons in hidden layer** | **1** | **2** | **3** |
| --- | --- | --- | --- |
| **Threshold** | -6.9682 | 6.3212 | -1.3805 |

**The weights of connection between the hidden layer and the output layer**

| **ID of neurons in hidden layer** | **1** | **2** | **3** |
| --- | --- | --- | --- |
| **Weight of connection** | -0.2507 | 0.2912 | 0.5216 |

**Threshold of neuron in output layer**

| **ID of neurons in hidden layer** | **1** |
| --- | --- |
| **Threshold** | -0.8850 |
